# Patient characteristics modify the association between changes in mineral metabolism parameters and mortality in a nationwide hemodialysis cohort study

**DOI:** 10.1038/s41598-025-92359-0

**Published:** 2025-03-08

**Authors:** Shunsuke Goto, Takayuki Hamano, Masatomo Taniguchi, Masanori Abe, Kosaku Nitta, Shinichi Nishi, Hideki Fujii

**Affiliations:** 1 Committee of the Renal Data Registry, Japanese Society for Dialysis Therapy, Tokyo, Japan; 2 Division of Nephrology and Kidney Center, Kobe University Graduate School of Medicine, Kobe, Japan; 3 Department of Nephrology, Nagoya City University, Graduate School of Medical Sciences, Nagoya, Japan; 4 Department of Nephrology, Osaka University Graduate School of Medicine, Suita, Japan; 5 Fukuoka Renal Clinic, Fukuoka, Japan; 6 Division of Nephrology, Hypertension and Endocrinology, Department of Internal Medicine, Nihon University School of Medicine, Tokyo, Japan; 7 Department of Nephrology, Tokyo Women’s Medical University, Tokyo, Japan

**Keywords:** Calcium, Diabetes mellitus, Hemodialysis, Performance status, Phosphate, Renal replacement therapy, Parathyroid diseases

## Abstract

In hemodialysis patients, it remains unclear whether patient characteristics influence the clinical impacts of changes in serum mineral metabolism parameters on mortality. In this 9-year cohort study, we investigated the associations between the changes in calcium/phosphate levels and all-cause mortality using a time-dependent approach after adjustment for potential confounders in groups stratified by performance status (PS), a history of atherosclerotic cardiovascular disease (ACVD), or diabetic nephropathy (DN). In patients with baseline serum calcium levels of 9.5–<10.0 mg/dL, increases in serum calcium levels were associated with higher mortality exclusively in patients with PS Grade 0. In the same baseline calcium range, a significant association was observed between reduced serum calcium levels and lower mortality only in patients with a history of ACVD or DN. Similarly, in patients with baseline serum phosphate levels of 5.0–<5.5 mg/dL, reduced serum phosphate levels were associated with lower mortality only in those with PS Grade 0, a history of ACVD or DN. These findings indicate that PS should be considered in treating mild hypercalcemia or hyperphosphatemia in hemodialysis patients. Moreover, stringent management of hypercalcemia and hyperphosphatemia in patients with a history of ACVD or DN might be associated with a better prognosis.

**Supplementary Information:**

The online version contains supplementary material available at 10.1038/s41598-025-92359-0.

## Introduction

Numerous observational studies have demonstrated an association between elevated serum calcium/phosphate levels and an enhanced risk of mortality in patients undergoing hemodialysis^1–3^. Increased calcium and phosphate load have been linked to accelerated vascular and cardiac valve calcification in hemodialysis patients^4–6^, thereby indicating hypercalcemia and hyperphosphatemia to be significant risk factors for mortality in this group of patients. The Kidney Disease: Improving Global Outcome (KDIGO) guidelines have suggested that elevated levels of calcium and phosphate should be lowered toward the normal ranges, based on this evidence^7^. Although a few previous observational studies have demonstrated that a reduction in these parameters is associated with a lower mortality risk^8,9^, the evidence is limited. Randomized controlled trials can prove the clinical impacts of reducing serum calcium or phosphate levels; however, currently there is limited evidence available^10,11^.

In patients undergoing hemodialysis, the association between serum phosphate levels and mortality depends on patient characteristics^12–14^. The performance status (PS) of the patients undergoing hemodialysis modified the association between serum phosphate levels and mortality^12^. Our previous study has demonstrated that hemodialysis patients with a history of atherosclerotic cardiovascular disease (ACVD) or diabetic nephropathy (DN) are more likely to benefit from reduced serum phosphate levels^13^. Therefore, the association of changes in phosphate levels with mortality may differ across groups stratified by PS or a history of ACVD or DN.

This study used the database of the Japanese Society for Dialysis Therapy Renal Data Registry (JRDR) to examine the association between changes in serum calcium/phosphate levels and mortality in hemodialysis patients using a time-dependent approach, stratified by PS, a history of ACVD, or a renal etiology of DN.

## Methods

### Data source, study population, and study design

Data from our previous studies^2,13^ was used in this 9-year cohort study. The JRDR is a nationwide registry, its data has been constituted through questionnaires distributed to all dialysis facilities in Japan by the Japanese Society for Dialysis Therapy (JSDT), the response rate for which was 95%. A more detailed description of the survey protocol can be found elsewhere^15^.

Patients undergoing maintenance hemodialysis or hemodiafiltration three times per week at the end of 2009 constituted the study population. Inclusion criteria for the study were: patients aged 18 years or older; treatment durations of 3–6 h per session; a dialysis vintage of one year or more; having data on calcium, phosphate, intact or whole parathyroid hormone (PTH), and albumin at the end of 2009 (to serve as baseline); and having at least one or more data on calcium, phosphate, intact or whole PTH, and albumin that had been collected after 2010. Patients receiving combined therapy with PD and those with implausible outcome data were excluded from the study (Fig. S1). The Ethics Committee of the JSDT approved the study protocol and waived the need for informed consent since the data were anonymized prior to being transferred to the investigators (approval number 54). Patients were provided with the option to opt out of participation. The study was conducted in accordance with the principles of the Declaration of Helsinki.

### Definitions

The following formula was used for correcting the serum calcium levels: Corrected calcium = serum calcium (mg/dL) + [4 - serum albumin (g/dL)], for serum albumin levels, which were less than 4 g/dL^16^. If whole PTH assays were used, the whole PTH levels were multiplied by 1.7 to obtain equivalent values for the intact PTH assay^17^. The PS was classified in accordance with the Eastern Cooperative Oncology Group Performance Status classification^18^.

### Statistical analysis

Continuous variables with normal distributions have been expressed in terms of mean and standard deviation, whereas the skewed distributions have been expressed as median and interquartile range (IQR), and the categorical variables as number and percentage.

The relationship between changes in serum calcium/phosphate levels and all-cause mortality was investigated using the multivariate Cox proportional hazard models. Changes in serum calcium and phosphate levels were calculated by deducting the baseline values from the mean serum values of each subsequent year, in accordance with a previous study (Fig. S2A)^8^. The analyses were stratified based on baseline serum calcium or phosphate levels (calcium: <8.4, 8.4–<9.0, 9.0–<9.5, 9.5–<10.0, or ≥ 10.0 mg/dL; phosphate: <3.5, 3.5–<5.0, 5.0–<5.5, 5.5–<6.0, or ≥ 6.0 mg/dL), given that the association between the changes in serum calcium/phosphate levels and mortality were subject to variations based on baseline^8,9^. The cut-off values were determined based on the distribution of participants and the recommended ranges according to the Japanese guidelines^17^. We investigated the association in the whole patients subjected for analyses and subgroups stratified by PS (Grade 0, Grade 1, or Grade 2–4), a history of ACVD, or a renal etiology of DN. Moreover, the proportion of all-cause death and cardiovascular disease (CVD) death, the relationship of TA phosphate and calcium with mortality due to heart failure, and the correlation between changes of serum phosphate levels and those of serum albumin levels in groups stratified by PS were investigated to better understand the mechanism of variations across groups stratified by PS. The same method as that used for calculating the changes in calcium or phosphate was used to calculate the changes in serum albumin levels.

The association of the changes in serum intact PTH levels with all-cause mortality were also investigated. Changes in serum intact PTH levels were determined as indicated in a previous study, by dividing the mean serum value of each year by the baseline values (Fig. S2B)^19^. Since patients with PTH levels ≥ 600 pg/ml accounted for only 1.9% of the total enrolled patients, the analyses were stratified based on the baseline serum intact PTH levels (< 60, 60–<120, 120–<180, 180–<240, or 240–<600 pg/mL).

Restricted cubic spline models were used to examine the nonlinear associations between changes in serum calcium, phosphate, and intact PTH levels and mortality. In order to mitigate the influence of outliers, values above the 99th percentile and below the 1st percentile of changes in serum calcium, phosphate, and intact PTH levels were excluded. The Akaike Information Criterion was applied for determining the number of knots in each model.

Covariates in these analyses included age, sex, dialysis vintage, dialysis time, dialysis modality (hemodialysis or hemodiafiltration), body mass index (BMI), Kt/V, normalized protein catabolic rate (nPCR), primary cause of renal failure (glomerulonephritis, DN, hypertension, polycystic kidney disease, others, or unknown), history of myocardial infarction, cerebral hemorrhage, cerebral infarction, amputation, hip fracture, parathyroidectomy (PTx), or percutaneous ethanol injection therapy (PEIT), albumin, creatinine, C-reactive protein (CRP), hemoglobin, magnesium, alkaline phosphatase (ALP), chronic kidney disease-mineral and bone disorder (CKD-MBD) related medications (calcium carbonate, sevelamer chloride, lanthanum carbonate, other phosphate binders, oral vitamin D receptor activator (VDRA), intravenous VDRA, and cinacalcet), dialysate calcium concentration (< 2.75, 2.75 to < 3.0, ≥ 3.0 mEq/L, and acetate-free), and PS (0–4). Among these variables, the covariates including age, dialysis time, BMI, Kt/V, PCR, history of myocardial infarction, cerebral hemorrhage, cerebral infarction, amputation, albumin, creatinine, CRP, and hemoglobin were considered to be the time-varying covariates. For calcium, phosphate, and intact PTH, the analyses were adjusted for the baseline values of the targeted parameter and the time-varying covariates of the other two parameters when analyzing the associations for one parameter. When the number of hemodialysis sessions per week was changed to something other than three, the time-varying covariates were treated as missing values. CRP was log-transformed to normalize its distribution. Based on the U-shaped associations between the variables, serum calcium, phosphate, magnesium, and intact PTH levels and mortality, serum calcium, phosphate, and intact PTH levels were divided into deciles, whereas serum magnesium levels were divided into quintiles^2,20^.

P value < 0.05 was considered statistically significant. In the comparison of each baseline variable between two groups, standardized differences ≥ 10% indicate an imbalance between variables. The nonparametric test for trend developed by Cuzick was used for assessing the trends across the three groups. The Stata/MP 14.2 software for Windows (Stata, College Station, TX, USA) was used for performing all statistical analyses.

## Results

The study population included a total of 68,354 hemodialysis patients. Patients with poor PS were generally older, predominantly female, had shorter dialysis durations, lower BMI and nPCR, reduced hemodiafiltration, and less frequent CKD-MBD-related medication and high-calcium dialysate use (Table 1). They were more likely to have a history of DN, CVD, and fractures, along with lower levels of serum phosphate, PTH, albumin, creatinine, hemoglobin, and magnesium, and higher levels of serum calcium, CRP, and ALP. Patients with ACVD were older, predominantly male, had shorter dialysis durations, lower Kt/V and nPCR, and lesser use of calcium carbonate, sevelamer, lanthanum carbonate, and cinacalcet (Table 2). They were also characterized by a greater prevalence of DN, cerebral hemorrhage, and fractures, and exhibited reduced serum phosphate, albumin, creatinine, and magnesium levels, elevated CRP levels and poorer PS. Patients with DN were older, predominantly male, and the other characters included shorter dialysis durations, lower Kt/V, higher BMI, less hemodiafiltration, along with reduced sevelamer, intravenous VDRA, and cinacalcet usage. These patients also had a higher prevalence of ACVD and lower serum calcium, PTH, and creatinine levels.

**Table 1:** Baseline characteristics of the groups stratified based on PS.

	Performance status			*P* for trend
	Grade 0 (*N* = 33,934)	Grade 1 (*N* = 21,460)	Grade 2–4 (*N* = 12,960)	
Age (years)	62.2 ± 12.0	66.9 ± 11.3	73.2 ± 10.7	< 0.001
Men (%)	66.3	58.0	53.0	< 0.001
Dialysis vintage (month)	81 (42–144)	78 (40–144)	68 (36–123)	< 0.001
Dialysis time (hour/week)	12.2 ± 1.4	12.1 ± 1.4	11.6 ± 1.4	< 0.001
Hemodiafiltration (%)	7.7	7.4	6.3	< 0.001
BMI (kg/m^2^)	21.7 ± 3.8	21.4 ± 3.9	20.6 ± 4.7	< 0.001
Kt/V	1.44 ± 0.29	1.46 ± 0.29	1.44 ± 0.29	0.058
nPCR (g/kg/day)	0.91 ± 0.17	0.89 ± 0.17	0.84 ± 0.17	< 0.001
Cause of kidney failure (%)				
Glomerulonephritis	46.0	39.9	29.7	< 0.001
Diabetes	27.9	34.2	45.1	< 0.001
Hypertension	5.5	7.0	9.0	< 0.001
Polycystic kidney disease	4.5	3.7	2.1	< 0.001
Others	7.1	7.0	7.7	0.105
Unknown	8.9	8.3	6.4	< 0.001
Past history (%)				
Myocardial infarction	6.9	9.7	12.2	< 0.001
Cerebral hemorrhage	3.4	4.8	11.7	< 0.001
Cerebral infarction	9.7	15.3	32.4	< 0.001
Amputation	1.5	2.6	8.0	< 0.001
Fracture	0.9	2.0	7.3	< 0.001
PTx	7.4	7.6	4.2	< 0.001
PEIT	1.6	1.6	0.9	< 0.001
Laboratory test				
Corrected calcium (mg/dL)	9.3 ± 0.8	9.3 ± 0.8	9.4 ± 1.0	0.003
Phosphate (mg/dL)	5.4 ± 1.4	5.2 ± 1.4	4.8 ± 1.4	< 0.001
Intact PTH (pg/mL)	131 (70–214)	126 (66–207)	112 (56–190)	< 0.001
Albumin (g/dL)	3.8 ± 0.3	3.7 ± 0.3	3.5 ± 0.4	< 0.001
Creatinine (mg/dL)	11.7 ± 2.7	10.7 ± 2.5	9.0 ± 2.4	< 0.001
CRP (mg/dL)	0.1 (0.0–0.2)	0.1 (0.0–0.3)	0.1 (0.0–0.5)	< 0.001
Hemoglobin (g/dl)	10.8 ± 1.1	10.7 ± 1.2	10.5 ± 1.3	< 0.001
Magnesium (mg/dL)	2.7 ± 0.5	2.6 ± 0.5	2.5 ± 0.5	< 0.001
ALP (IU/L)	244 ± 122	261 ± 127	287 ± 151	< 0.001
Medication (%)				
Calcium carbonate	68.0	65.9	53.3	< 0.001
Sevelamer	38.9	33.4	19.5	< 0.001
Lanthanum carbonate	17.6	15.4	9.0	< 0.001
Other phosphate binders	4.6	3.7	2.2	< 0.001
Oral VDRA	39.3	40.6	36.2	< 0.001
Intravenous VDRA	32.7	30.9	23.7	< 0.001
Cinacalcet	17.0	14.4	8.5	< 0.001
Dialysate calcium (%)				
<2.75 mEq/L	36.8	38.4	38.7	< 0.001
2.75-<3.0 mEq/L	9.0	8.2	8.4	0.003
3.0- mEq/L	50.2	49.3	49.2	0.019
Acetate-free dialysis	3.9	4.1	3.8	0.727
Performance status (%)				
Grade 0	100.0	0.0	0.0	
Grade 1	0.0	100.0	0.0	
Grade 2	0.0	0.0	60.2	
Grade 3	0.0	0.0	26.3	
Grade 4	0.0	0.0	13.5	

PS: performance status; BMI: body mass index; nPCR: normalized protein catabolic rate; PEIT: percutaneous ethanol injection therapy; PTx: parathyroidectomy; PTH: parathyroid hormone; CRP: C-reactive protein; ALP: alkaline phosphatase; VDRA: vitamin D receptor activator.

**Table 2:** Baseline characteristics of the groups stratified based on a history of ACVD or a renal etiology of DN.

	ACVD (*N* = 17,413)	No ACVD (*N* = 50,941)	SD, %	DN (*N* = 22,673)	No DN (*N* = 45,681)	SD, %
Age (years)	69.6 ± 10.3	64.5 ± 12.6	44.4	66.6 ± 10.7	65.4 ± 12.9	10.1
Men (%)	66.8	59.3	15.5	67.2	58.2	18.7
Dialysis vintage (month)	71 (38–125)	80 (41–146)	16.2	54 (31–88)	97 (48–173)	77.4
Dialysis time (hour/week)	11.9 ± 1.4	12.1 ± 1.4	10.9	11.9 ± 1.3	12.1 ± 1.4	14.6
Hemodiafiltration (%)	6.7	7.5	3.1	5.1	8.5	13.5
BMI (kg/m^2^)	21.4 ± 4.7	21.4 ± 3.8	0.6	22.3 ± 4.1	21.0 ± 3.9	34.2
Kt/V	1.41 ± 0.28	1.46 ± 0.29	17.2	1.37 ± 0.27	1.49 ± 0.29	43.8
nPCR (g/kg/day)	0.86 ± 0.17	0.90 ± 0.17	24.1	0.85 ± 0.17	0.91 ± 0.17	33.5
Cause of kidney failure (%)						
Glomerulonephritis	29.9	44.8	31.3	0.0	61.4	
Diabetes	47.7	28.2	40.9	100.0	0.0	
Hypertension	7.9	6.2	6.7	0.0	9.9	
Polycystic kidney disease	2.8	4.1	7.4	0.0	5.6	
Others	6.0	7.6	6.3	0.0	10.8	
Unknown	5.8	9.1	12.4	0.0	12.3	
Past history (%)						
Myocardial infarction	34.4	0.0		13.1	6.6	22.0
Cerebral hemorrhage	8.9	4.3	18.7	5.0	5.7	3.1
Cerebral infarction	62.0	0.0		21.7	12.9	23.4
Amputation	12.0	0.0		6.8	1.2	28.9
Fracture	3.7	2.0	10.1	2.8	2.3	3.2
PTx	4.9	7.5	10.8	1.4	9.6	36.8
PEIT	1.2	1.5	2.5	0.5	1.9	13.0
Laboratory test						
Corrected calcium (mg/dL)	9.3 ± 0.9	9.3 ± 0.9	2.4	9.2 ± 0.8	9.4 ± 0.9	20.4
Phosphate (mg/dL)	5.1 ± 1.4	5.3 ± 1.4	14.4	5.1 ± 1.4	5.3 ± 1.4	7.6
Intact PTH (pg/mL)	121 (63–198)	127 (67–210)	7.1	114 (60–187)	132 (70–218)	19.3
Albumin (g/dL)	3.7 ± 0.4	3.8 ± 0.4	24.2	3.7 ± 0.4	3.7 ± 0.4	4.5
Creatinine (mg/dL)	10.1 ± 2.6	11.1 ± 2.7	38.3	10.2 ± 2.6	11.2 ± 2.8	39.2
CRP (mg/dL)	0.1 (0.0–0.4)	0.1 (0.0–0.2)	11.9	0.1 (0.0–0.3)	0.1 (0.0–0.3)	1.6
Hemoglobin (g/dl)	10.6 ± 1.2	10.7 ± 1.2	5.0	10.7 ± 1.2	10.7 ± 1.2	3.2
Magnesium (mg/dL)	2.6 ± 0.5	2.7 ± 0.5	14.0	2.6 ± 0.5	2.6 ± 0.5	2.4
ALP (IU/L)	263 ± 119	256 ± 134	5.8	256 ± 123	258 ± 134	1.2
Medication (%)						
Calcium carbonate	60.8	65.8	10.4	65.3	64.1	2.5
Sevelamer	28.2	35.3	15.4	27.6	36.4	19.1
Lanthanum carbonate	12.4	16.2	10.8	13.4	16.2	7.8
Other phosphate binders	3.3	4.1	4.1	3.1	4.2	6.0
Oral VDRA	38.1	39.5	2.8	40.0	38.7	2.8
Intravenous VDRA	27.8	31.3	7.7	23.3	34.0	23.7
Cinacalcet	11.9	15.5	10.7	7.3	18.2	33.1
Dialysate calcium (%)						
<2.75 mEq/L	37.3	37.8	1.0	38.5	37.2	2.6
2.75-<3.0 mEq/L	8.5	8.7	0.6	7.9	9.0	4.0
3.0- mEq/L	50.2	49.6	1.2	49.8	49.7	0.0
Acetate-free dialysis	4.0	3.9	0.5	3.8	4.0	0.9
Performance status (%)						
Grade 0	34.1	55.0	43.0	41.8	53.5	23.6
Grade 1	31.8	31.2	1.3	32.4	30.9	3.2
Grade 2	18.8	8.9	28.8	15.1	9.6	16.7
Grade 3	9.5	3.4	25.0	7.2	3.9	14.4
Grade 4	5.8	1.5	23.3	3.6	2.1	9.0

ACVD: atherosclerotic cardiovascular disease; DN: diabetic nephropathy; SD: standardized difference; BMI: body mass index; nPCR: normalized protein catabolic rate; PEIT: percutaneous ethanol injection therapy; PTx: parathyroidectomy; PTH: parathyroid hormone; CRP: C-reactive protein; ALP: alkaline phosphatase; VDRA: vitamin D receptor activator.

A total of 26,364 deaths occurred during the median observational period of 108 (IQR 55–108) months. In restricted cubic spline models, patients with normal or high calcium levels at baseline (≥ 8.4 mg/dL) were associated with a higher mortality risk following an increase in serum calcium levels from baseline values, whereas patients with low baseline calcium levels exhibited no such associations (Fig. S3). In patients with baseline serum calcium levels ≥ 9.5 mg/dL, significant associations were found between decreased serum calcium levels and lower mortality.

**Fig. 1:**
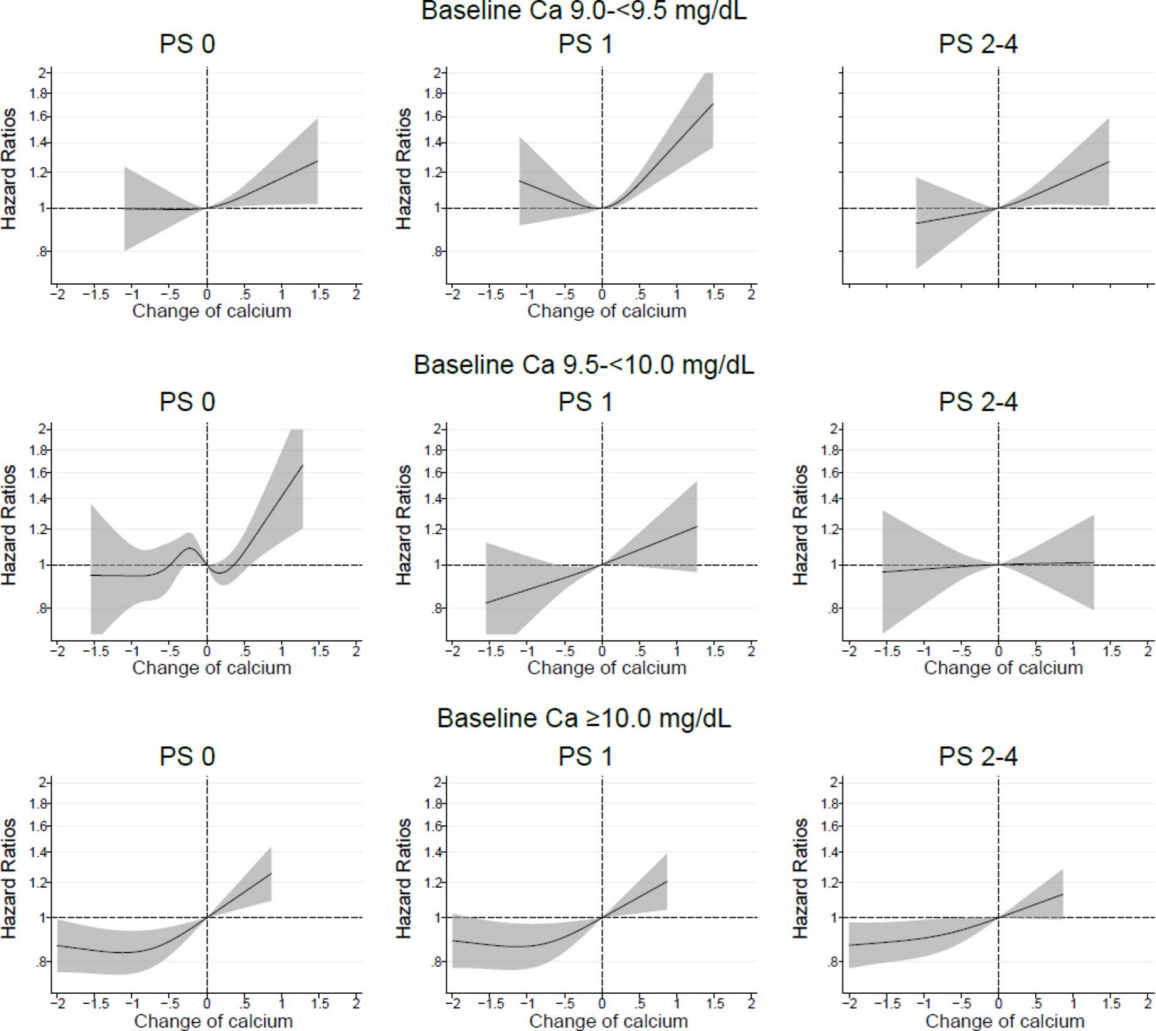
In patients with baseline serum calcium (Ca) levels 9.0-<9.5, 9.5-<10.0, or ≥ 10.0 mg/dL, relationship between changes in serum Ca levels from baseline values and all-cause mortality in patients stratified by performance status (PS).

In analyses stratified by PS, similar trends were observed among patients with baseline serum calcium levels of 9.0–<9.5 or ≥ 10.0 mg/dL. In patients with serum calcium levels ranging from 9.5–<10.0 mg/dL, elevated serum calcium levels were associated with mortality risk in patients with PS Grade 0, whereas the association was attenuated in patients with higher PS Grade (Fig. 1).

**Fig. 2:**
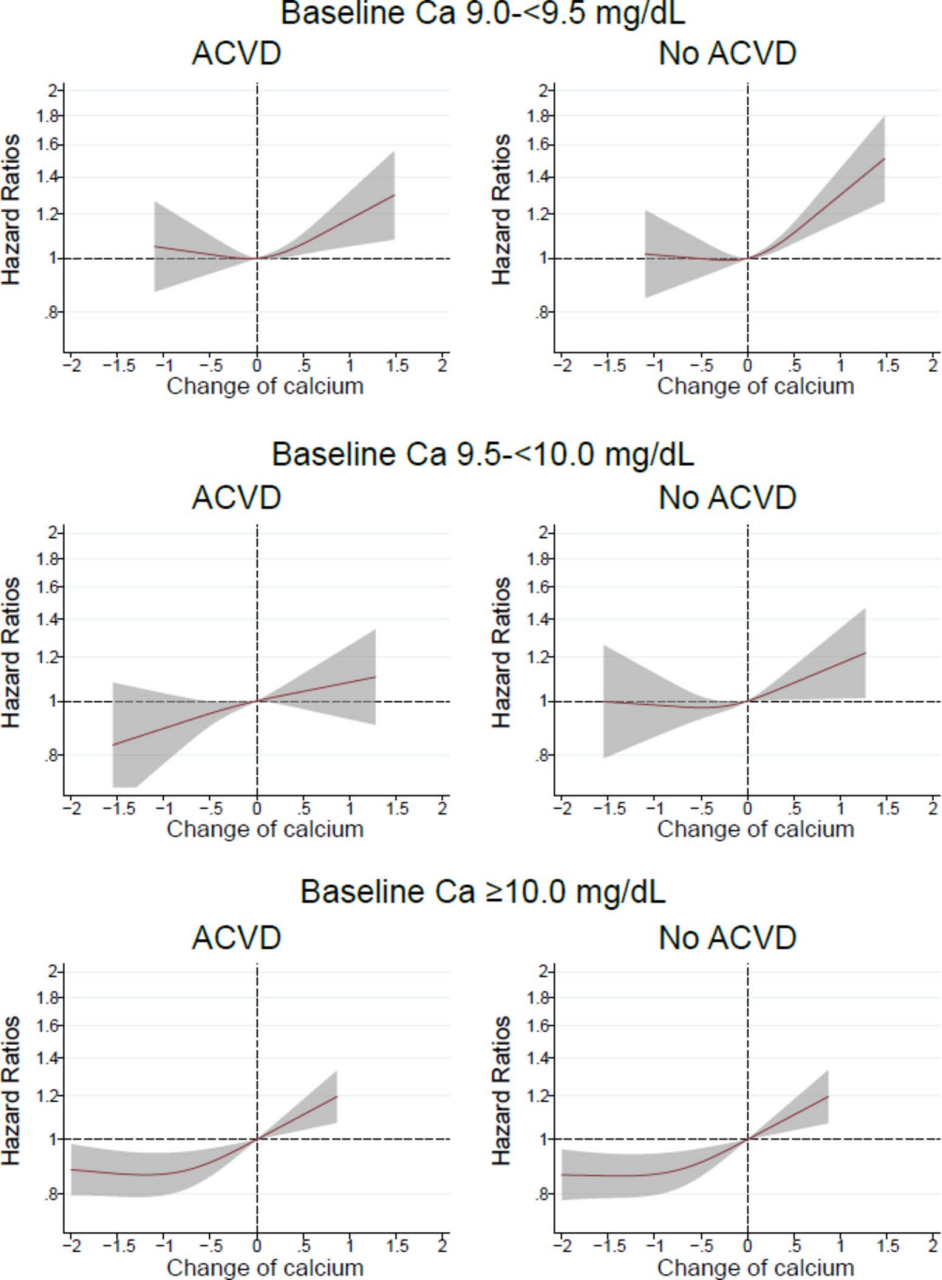
In patients with baseline serum calcium (Ca) levels 9.0-<9.5, 9.5-<10.0, or ≥ 10.0 mg/dL, relationship between changes in serum Ca levels from baseline values and all-cause mortality in patients with or without a history of atherosclerotic cardiovascular disease (ACVD).

**Fig. 3:**
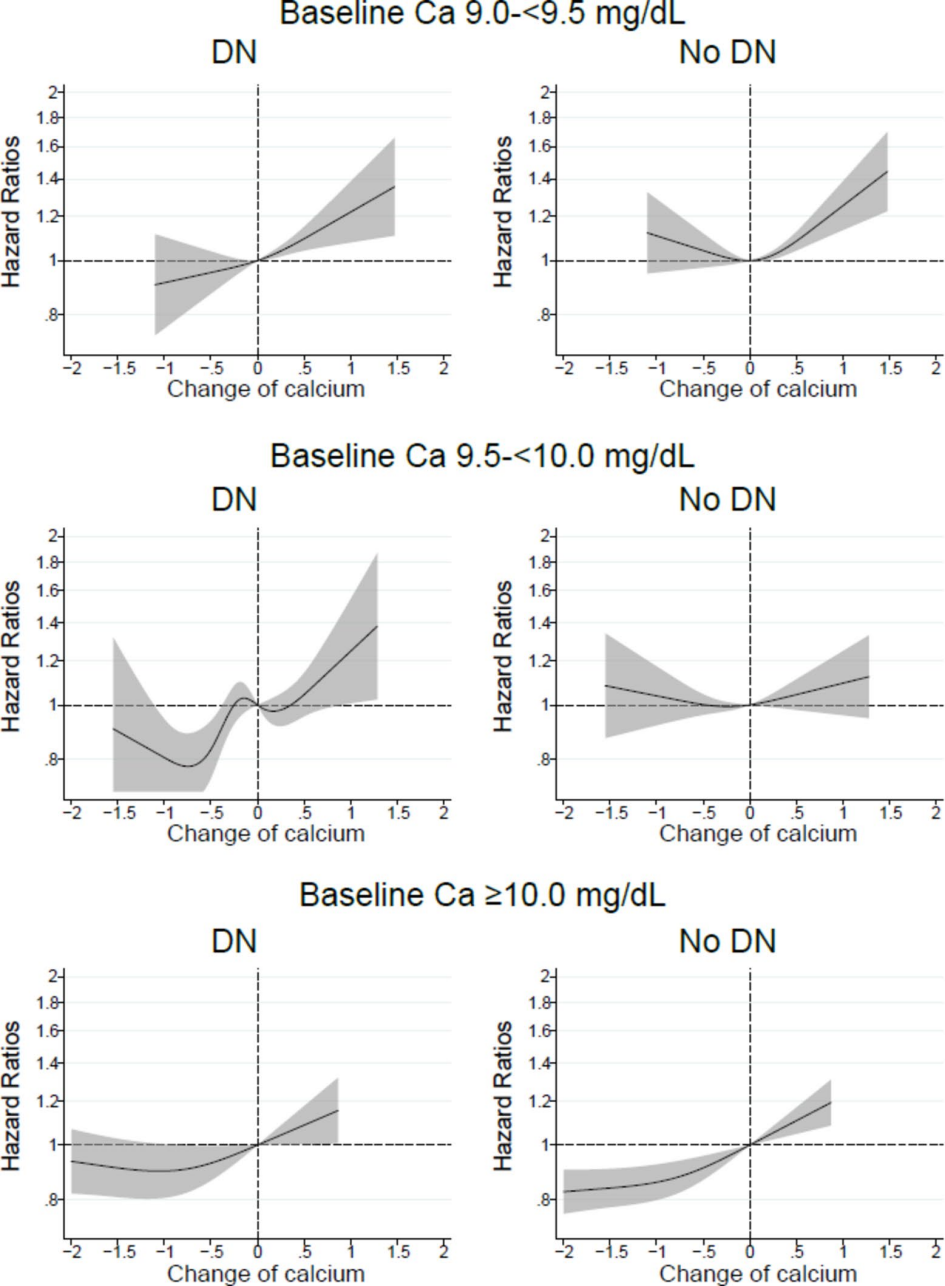
In patients with baseline serum calcium (Ca) levels 9.0–<9.5, 9.5–<10.0, or ≥ 10.0 mg/dL, relationship between changes in serum Ca levels from baseline values and all-cause mortality in patients with or without a renal etiology of diabetic nephropathy (DN).

The association between changes in calcium levels and mortality was modified by a history of ACVD or renal etiology of DN. In patients with baseline serum calcium levels of 9.5–<10.0 mg/dL, patients with ACVD or DN exhibited lower mortality due to decreased calcium levels, whereas no such associations were observed in patients without ACVD or DN (Figs. 2, 3).

Regarding phosphate, in patients with baseline serum phosphate levels ≥ 3.5 mg/dL, elevated serum phosphate levels from baseline were associated with higher mortality (Fig. S4). In patients with baseline serum phosphate levels < 3.5 mg/dL, phosphate level increases less than 0.5 mg/dL were associated with lower mortality, whereas increases > 1.2 mg/dL were associated with higher mortality. In patients with baseline serum phosphate levels ≥ 5.0 mg/dL, decreased serum phosphate levels were associated with lower mortality, whereas in patients with baseline serum phosphate levels < 3.5 mg/dL, decreased phosphate levels were associated with higher mortality.

**Fig. 4:**
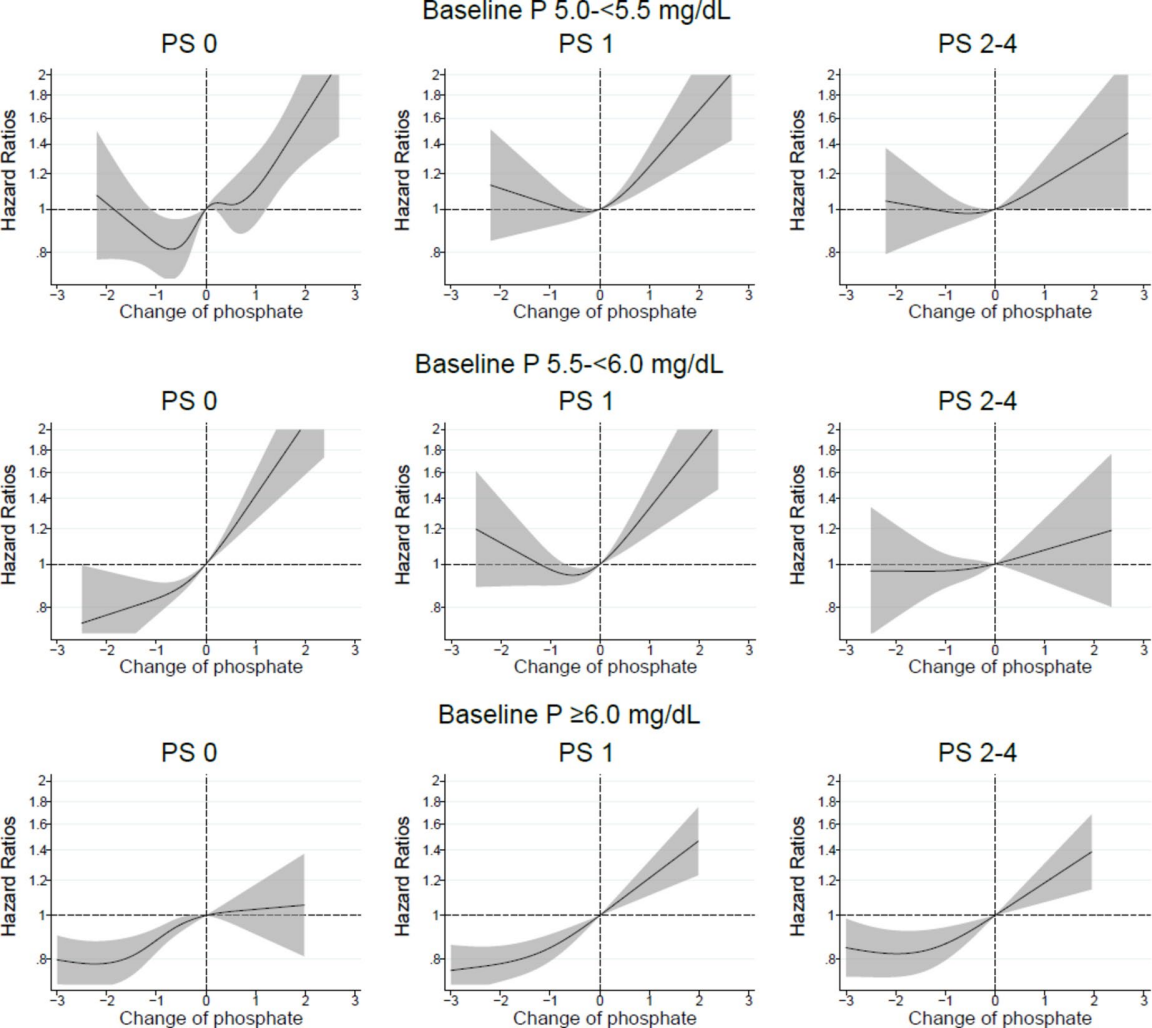
In patients with baseline serum phosphate (P) levels of 5.0–<5.5, 5.5–<6.0, or ≥ 6.0 mg/dL, relationship between changes in serum P levels from baseline values and all-cause mortality in patients stratified by performance status (PS).

The association between changes in serum phosphate levels and all-cause mortality was modified by PS (Fig. 4). Increased phosphate levels were associated with higher mortality and decreased phosphate levels were associated with lower mortality in PS Grade 0 patients, with baseline serum phosphate levels of 5.0–<6.0 mg/dL. However, the associations became weaker in patients with poorer PS.

**Fig. 5:**
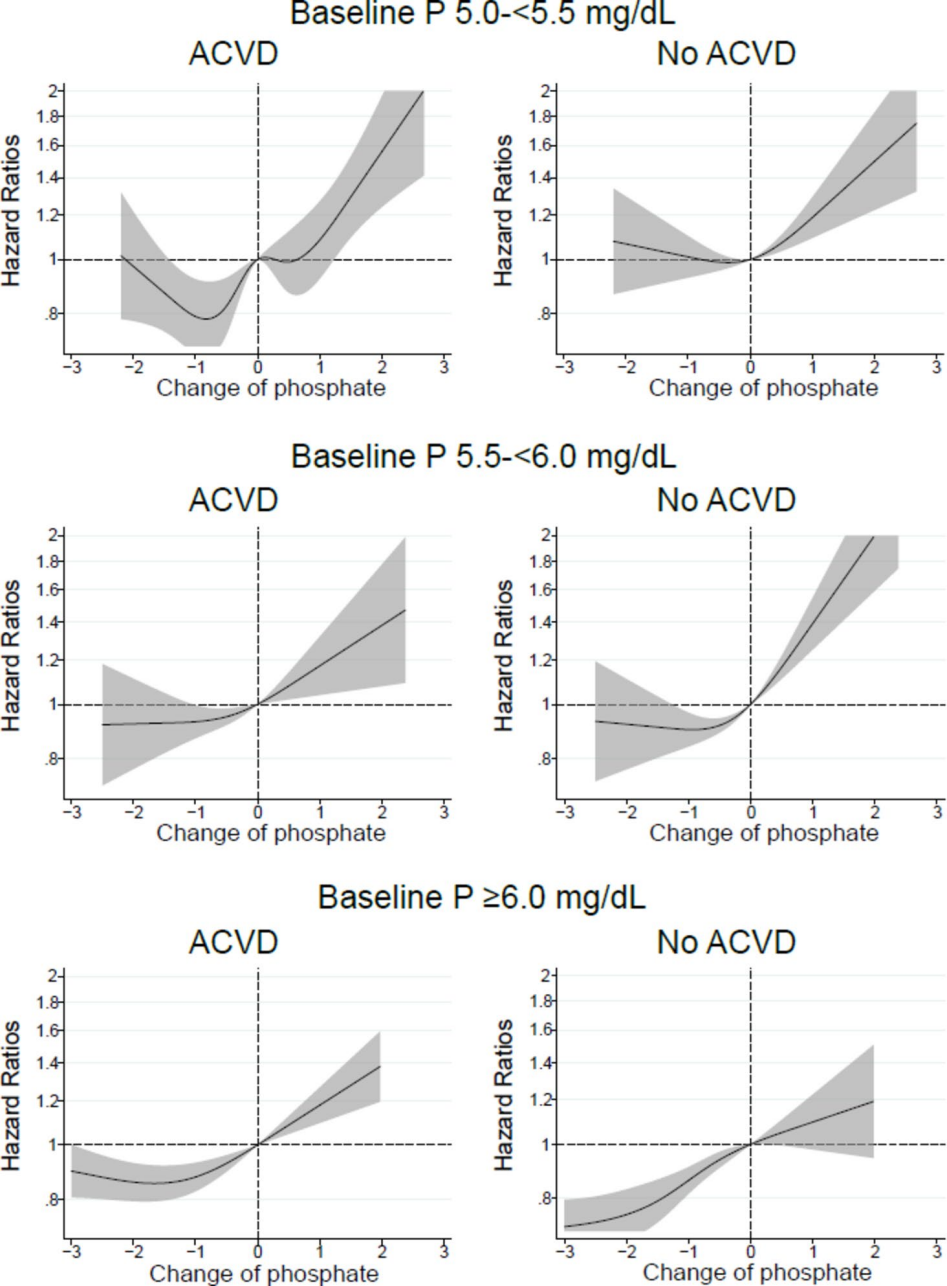
In patients with baseline serum phosphate (P) levels of 5.0–<5.5, 5.5–<6.0, or ≥ 6.0 mg/dL, relationship between changes in serum P levels from baseline values and all-cause mortality in patients with or without a history of atherosclerotic cardiovascular disease (ACVD).

**Fig. 6:**
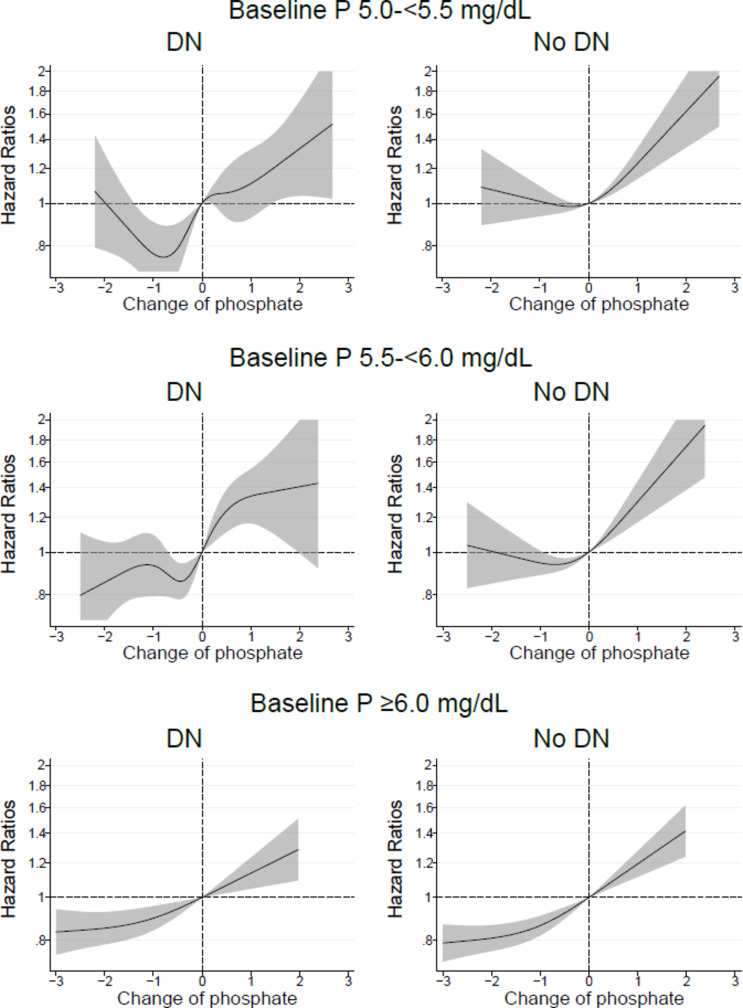
In patients with baseline serum phosphate (P) levels of 5.0–<5.5, 5.5–<6.0, or ≥ 6.0 mg/dL, relationship between changes in serum P levels from baseline values and all-cause mortality in patients with or without a renal etiology of diabetic nephropathy (DN).

 With regards to patients with a history of ACVD or renal etiology of DN, a lower mortality risk was observed following reductions in serum phosphate levels in patients with baseline serum phosphate levels ≥ 5.0 mg/dL; however, in patients without a history of ACVD or DN, and with baseline serum phosphate levels ≥ 5.5 mg/dL a significant association was observed between decreased serum phosphate levels and a lower mortality (Figs.  5, 6).

The proportion of CVD deaths was similar among the groups stratified by PS. Worse PS was associated with a higher proportion of heart failure related deaths (Fig. S5). With regards to the association of serum time-averaged (TA) calcium or phosphate levels with mortality due to heart failure, patients with PS Grade 0 demonstrated significant positive linear associations. However, with poorer PS, the associations were attenuated (Fig. S6). In patients with PS Grade 0, a reduction in serum phosphate levels was not associated with a reduction in serum albumin levels; however, in those with poor PS and baseline serum phosphate levels of 5.0–<6.0 mg/dL, reduced phosphate levels were associated with reduction in albumin levels (Fig. S7). In the case of patients with PS Grade 2–4 and baseline serum phosphate levels ≥ 6.0 mg/dL, decreases in phosphate levels > 1.0 mg/dL were associated with decreases in albumin.

In patients with serum PTH levels ≥ 180 pg/mL at baseline, PTH increases were associated with a higher mortality risk (Fig. S8). In patients with serum PTH levels < 60 pg/mL, 60–<120 pg/mL, and 120–<180 pg/mL, a higher mortality risk was observed, corresponding to 3.2-, 2.1-, and 1.5-fold increases from baseline values, respectively. In patients with baseline serum PTH levels < 240 pg/mL, decreases in serum PTH levels were not significantly associated with mortality. However, reductions in serum PTH levels were associated with a lower risk of mortality in patients with serum PTH levels 240–<600 pg/mL.

## Discussion

In this large cohort study based on a nationwide database, in patients with baseline calcium levels ≥ 10.0 mg/dL or baseline phosphate levels ≥ 6.0 mg/dL, decreases in serum calcium or phosphate levels were associated with a lower mortality risk, regardless of PS or a history of ACVD or DN. However, among patients with baseline calcium levels of 9.5–<10.0 mg/dL or baseline phosphate levels of 5.0–<6.0 mg/dL, the association between changes in serum calcium or phosphate levels and all-cause mortality was weaker in those with poor PS compared to those with good PS. Moreover, in patients with a history of ACVD or a renal etiology of DN and baseline calcium levels of 9.5–<10.0 mg/dL or phosphate levels of 5.0–<5.5 mg/dL, lower all-cause mortality associated with reduced serum calcium or phosphate levels was observed. In contrast, in patients without a history of ACVD or DN, reductions in serum calcium or phosphate levels were not associated with mortality at these baseline calcium or phosphate levels.

A previous study had demonstrated that poor PS attenuated the effect of hyperphosphatemia on all-cause mortality^12^. In our study, PS influenced the association of alterations in the serum phosphate as well as calcium levels with all-cause mortality. The precise mechanisms underlying this modification caused by PS remain unclear; however, the current study has demonstrated that the associations between serum calcium/phosphate levels and deaths caused by heart failure were weaker in patients with poor PS compared with patients with good PS. Moreover, patients with poor PS exhibit a greater proportion of deaths due to heart failure. Various factors are responsible for causing heart failure in patients with chronic kidney disease^21^. These findings suggest that factors other than calcium or phosphate could influence heart failure in hemodialysis patients with poor PS.

In this observational study, the factors responsible for reduced serum phosphate levels included either reduction due to interventions such as phosphate binders or non-intervention such as worsening nutritional status. A previous observational study had demonstrated that a worse prognosis was observed in hemodialysis patients with decreased protein intake and serum phosphate levels, contrary to patients with an increase in both parameters^22^. Among patients with poor PS (PS Grade ≥ 1) in our study, and in patients with baseline phosphate levels of 5.0–<6.0 mg/dL, decreased phosphate levels correlated with decreased albumin levels and were not associated with lower mortality; however, in patients with baseline phosphate levels ≥ 6.0 mg/dL, decrease in serum phosphate levels by 1.0 mg/dL, were not correlated with decreased albumin levels and were associated with lower mortality. These findings suggest that reduced serum phosphate levels without worsening nutrition might be associated with a lower risk of all-cause mortality even in patients with poor PS.

In this study, it was observed that hemodialysis patients with a history of ACVD or DN―particularly patients with lower baseline values―benefited to a greater extent from serum phosphate level reductions compared to those without a history of ACVD or DN. Our previous study had demonstrated linear associations between TA serum phosphate levels and all-cause mortality in hemodialysis patients with a history of ACVD or DN, whereas, the associations plateaued at serum phosphate levels below 5.0 mg/dL in patients without these histories^13^. In another study investigating non-dialysis patients, a stronger association was observed between elevated plasma phosphate levels and all-cause mortality in individuals with type 2 diabetes compared to those without a history of diabetes^23^. In vitro studies had demonstrated that compared with high-phosphate and normal-glucose medium, high-phosphate and high-glucose medium induced more severe calcium deposits in vascular smooth muscle cells, potentially through upregulation of a phosphate transporter or cellular-senescence-related factors^24–26^. In an animal study conducted on rats fed with high-phosphate diets, more severe vascular calcification was observed in rats with chronic kidney disease and diabetes mellitus as compared with rats having only chronic kidney disease^27^. Although fewer studies have examined the impact of hyperphosphatemia in groups stratified by a history of ACVD, individuals with the history of ACVD, characterized by more severe vascular calcification at baseline^28^, might tend to experience progression of vascular calcification, similar to diabetic patients, compared with those without ACVD. These findings suggest the significance of a stricter management of serum phosphate levels in hemodialysis patients with a history of ACVD or DN.

Similarly, for patients with serum calcium levels of 9.5–<10.0 mg/dL, only those with a history of ACVD or DN displayed an association between reduced calcium levels and a reduced mortality risk. Unfortunately, the association between serum calcium levels and mortality in hemodialysis patients stratified by a history of ACVD or DN has been seldom evaluated. The COSMOS study reported no significant effect modification by diabetes on the association between serum calcium levels and mortality, which is inconsistent with our findings^29^. The exact reason for this discrepancy is not clear. Since the sample size used in the current study had been larger compared with the COSMOS study, it might have made the detection of effect modification based on a history of ACVD or DN more feasible. High serum calcium levels are a well-established risk factor for vascular calcification in patients undergoing hemodialysis^4^. Therefore, stricter control of serum calcium levels, similar to phosphate levels, might confer greater benefits on hemodialysis patients with a history of ACVD or DN. Stricter calcium control can be achieved by reducing the use of calcium-based phosphate binders, optimizing VDRA therapy, and switching to a low-calcium dialysate.

Traditionally, excessive PTH suppression has been considered a cause of adynamic bone disease, leading to the progression of vascular calcification^30,31^. Therefore, the KDIGO guidelines have suggested a lower limit for PTH levels^7^. However, recent observational studies have reported that PTH levels below the suggested lower limit are not associated with an increased mortality risk in hemodialysis patients^2,19,32^. In this study, a decrease in serum PTH levels was not significantly associated with a higher mortality risk in patients whose baseline serum PTH levels were < 60 pg/mL. These findings suggest that we should reconsider whether a lower limit for serum PTH levels is necessary in these patients.

Our study found that decreases in serum PTH levels were associated with a lower mortality risk in patients with baseline PTH levels of 240–<600 pg/mL. The upper limit for PTH levels recommended in the Japanese guidelines (240 pg/mL) is lower than that specified in the KDIGO guidelines (nine times the upper normal limit for assay, i.e., 585 pg/mL)^7,17^. Although the optimal target range remains uncertain due to limited evidence, our findings suggest that lowering the upper limit of the KDIGO target range might be beneficial. Further research is needed to determine the most appropriate upper limit for serum PTH levels in patients undergoing hemodialysis.

The current Japanese guidelines, published in 2012, recommend a targeted range for serum phosphate levels between 3.5 and 6.0 mg/dL^17^, which is higher than that recommended by the KDIGO guidelines (the normal range)^7^. However, our study found that reduced serum phosphate levels were associated with a lower mortality risk in patients with baseline serum phosphate levels of 5.0–<6.0 mg/dL. A recent clinical trial demonstrated that stricter phosphate control (target: 3.5–<4.5 mg/dL) slowed the progression of vascular calcification compared with standard phosphate control (target: 5.0–<6.0 mg/dL)^11^. These findings suggest that lowering the upper limit of the target range for serum phosphate levels in the Japanese guidelines might be more beneficial, particularly for patients with good PS or a history of ACVD or DN. The primary strategies for achieving stricter phosphate control include increasing phosphate removal through dialysis, restricting dietary phosphate intake, using phosphate-lowering medications, and administering calcimimetics for patients with high PTH levels.

This study has several limitations. First, being an observational study, it could not establish causal relationships. Second, residual confounding may still be present despite adjusting for significant confounders. Third, the study population was limited to hemodialysis patients in Japan, and as such the findings may not be generalizable to other countries. Fourth, blood collection of most hemodialysis patients was performed at the beginning of the week in Japan^33^. Therefore, if blood is collected in the middle of the week, the results may be different. However, the large sample size and extended follow-up period are the strengths of the study, since they enabled the stratification of the patients into distinct ranges for serum calcium and phosphate levels.

In conclusion, our findings suggest the consideration of PS in treating mild hypercalcemia (Ca 9.5–<10.0 mg/dL) or hyperphosphatemia (P 5.0–<6.0 mg/dL) in hemodialysis patients. Furthermore, stricter management of hypercalcemia and hyperphosphatemia might lead to better prognosis in patients with a history of ACVD or DN.

## Electronic supplementary material

Below is the link to the electronic supplementary material.

Supplementary Material 1

## Data Availability

The data underlying this article were provided by the JSDT by permission. Data will be shared on request to the corresponding author with permission of the JSDT.
