# Supplementary material for: Patient characteristics modify the association between changes in mineral metabolism parameters and mortality in a nationwide hemodialysis cohort study
Source: Sci Rep. 2025 Mar 8;15:8089. doi: 10.1038/s41598-025-92359-0 (PMC11890868; doi:10.1038/s41598-025-92359-0)
Supplement: Supplementary file 1 — Supplementary Material 1 [file 41598_2025_92359_MOESM1_ESM.pdf]

Supplementary materials

Contents:

Figure S1. Flow diagram. JRDR, the Japanese Society for Dialysis Therapy Renal Data Registry; HD, hemodialysis; HDF, hemodiafiltration; Ca, calcium; P, phosphate; PTH, parathyroid hormone.

Figure S2: Outline of the analysis for (A) difference changes in calcium and phosphate and (B) percent changes in parathyroid hormone. T0, a value at baseline; T1, a value at one year after baseline; T2, a value at two years after baseline; T3, a value at three years after baseline.

Figure S3. Relationship between changes in serum calcium (Ca) levels from baseline values and all-cause mortality.

Figure S4. Relationship between changes in serum phosphate (P) levels from baseline values and all-cause mortality.

Figure S5. Proportion of (A) all-cause death and (B) cardiovascular death in subgroups stratified by performance status (PS).

Figure S6. Relationship of time-averaged (TA) serum calcium or phosphate levels with mortality due to heart failure in patients stratified by performance status (PS).

Figure S7. Relationship between changes in serum phosphate levels and those in serum albumin levels in patients stratified by performance status (PS).

Figure S8. Relationship between percent changes in serum intact parathyroid hormone (PTH) levels from baseline values and all-cause mortality.

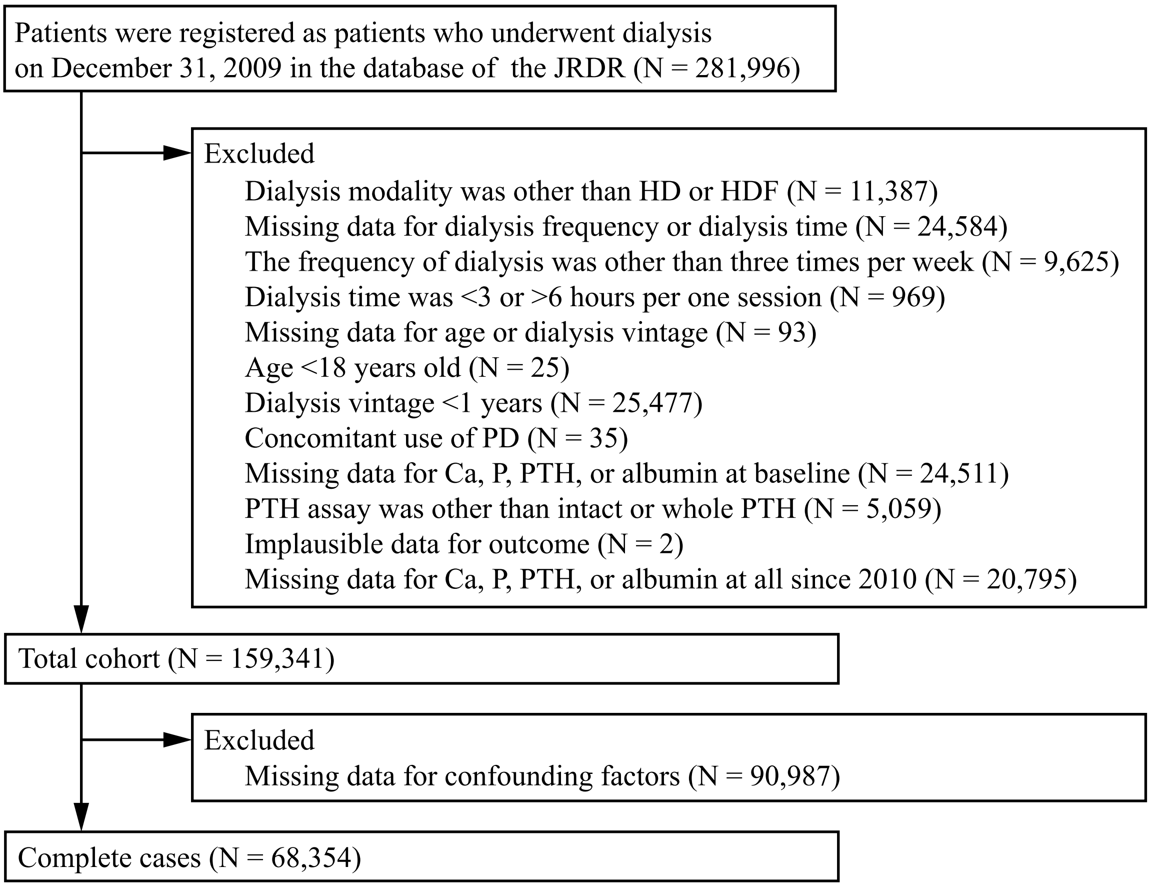

Figure S1. Flow diagram. JRDR, the Japanese Society for Dialysis Therapy Renal Data Registry; HD, hemodialysis; HDF, hemodiafiltration; Ca, calcium; P, phosphate; PTH, parathyroid hormone.

A

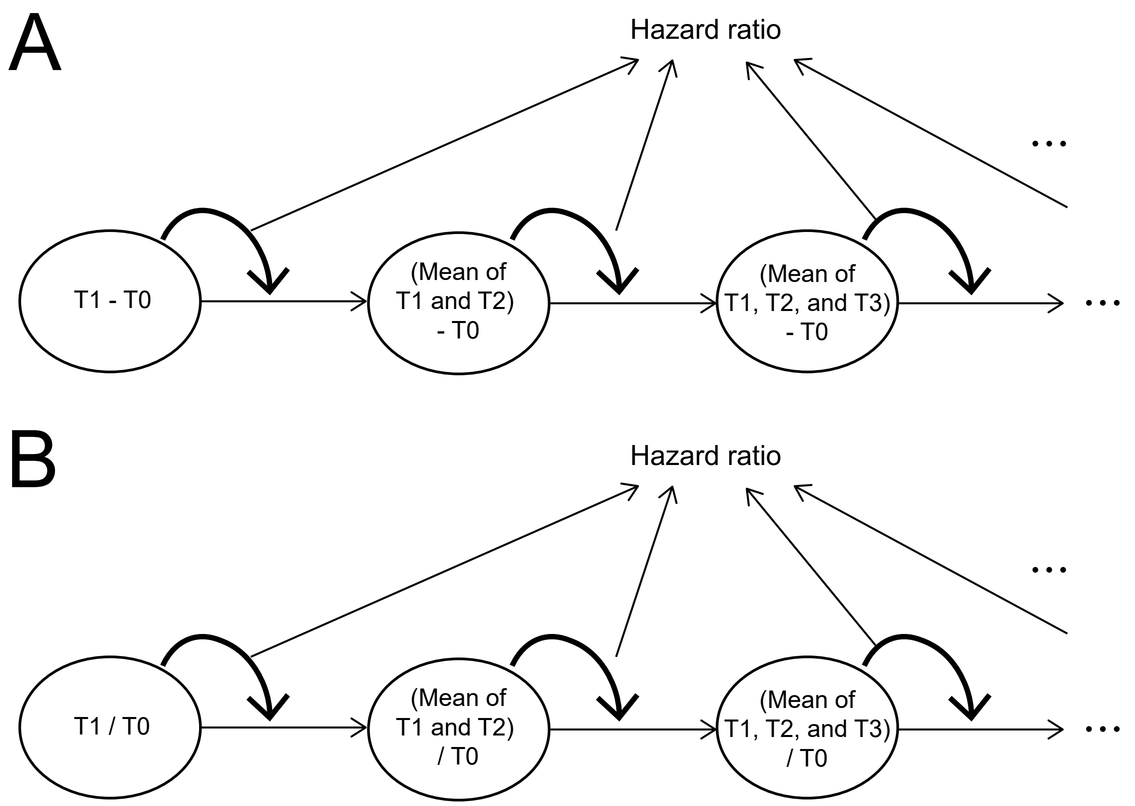

Figure S2. Outline of the analysis for (A) difference changes in calcium and phosphate and (B) percent changes in parathyroid hormone. T0, a value at baseline; T1, a value at one year after baseline; T2, a value at two years after baseline; T3, a value at three years after baseline.

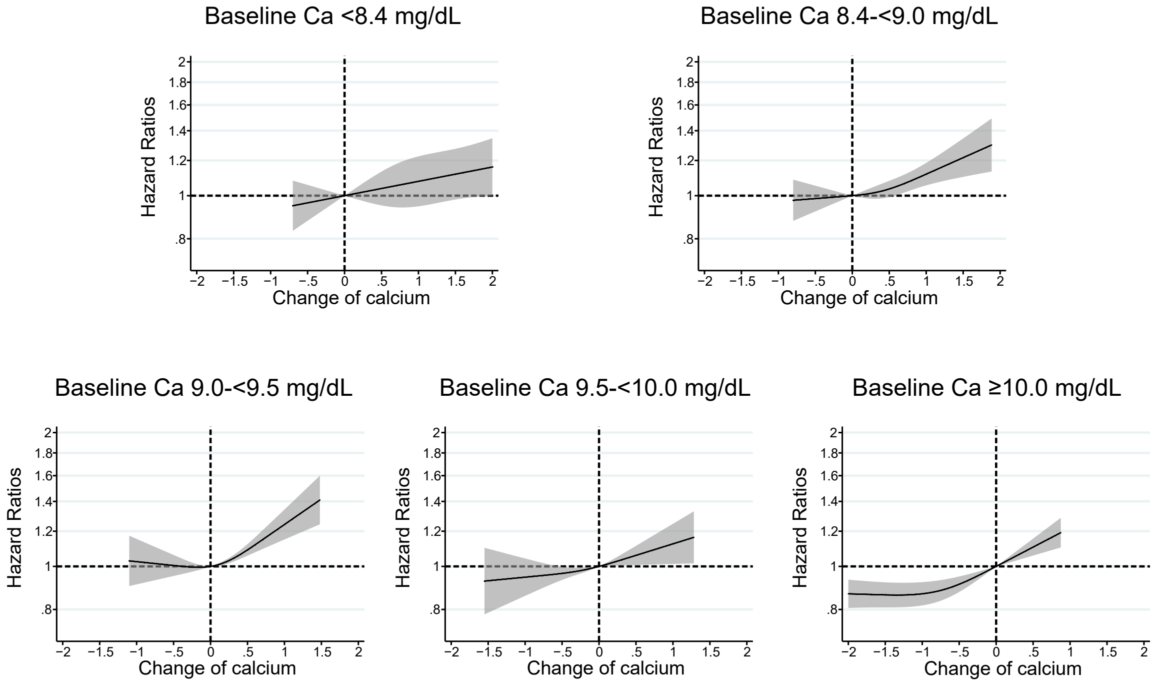

Figure S3. Relationship between changes in serum calcium (Ca) levels from baseline values and all-cause mortality.

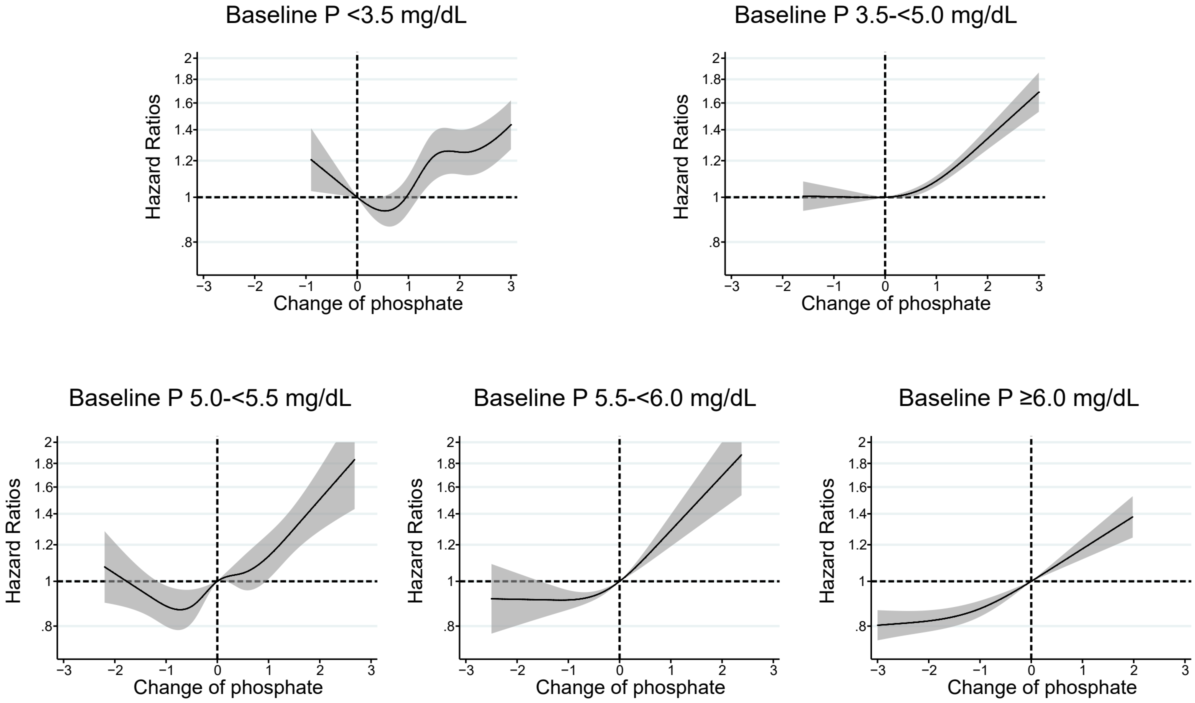

Figure S4. Relationship between changes in serum phosphate (P) levels from baseline values and all-cause mortality.

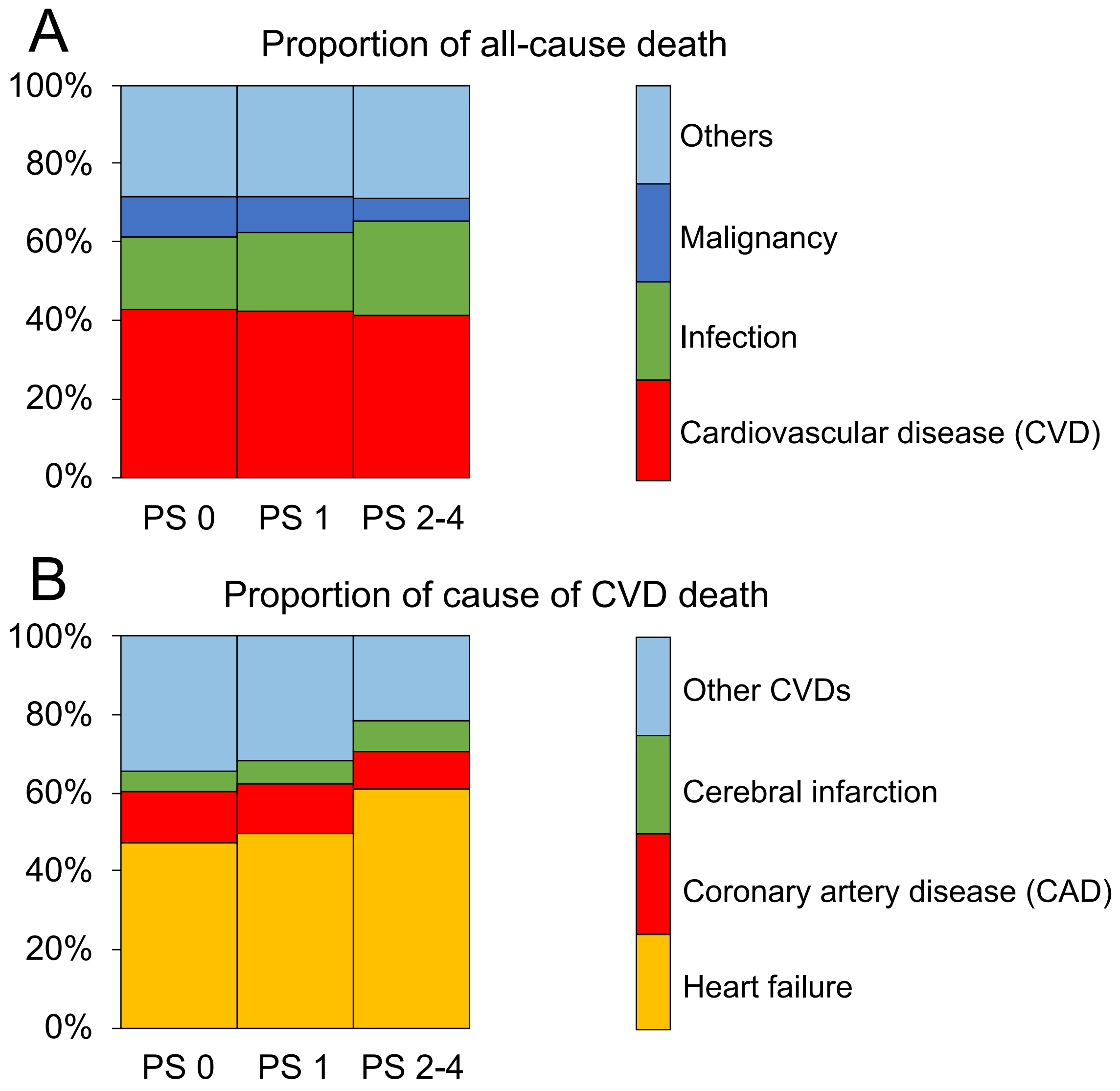

Figure S5. Proportion of (A) all-cause death and (B) cardiovascular death in subgroups stratified by performance status (PS).

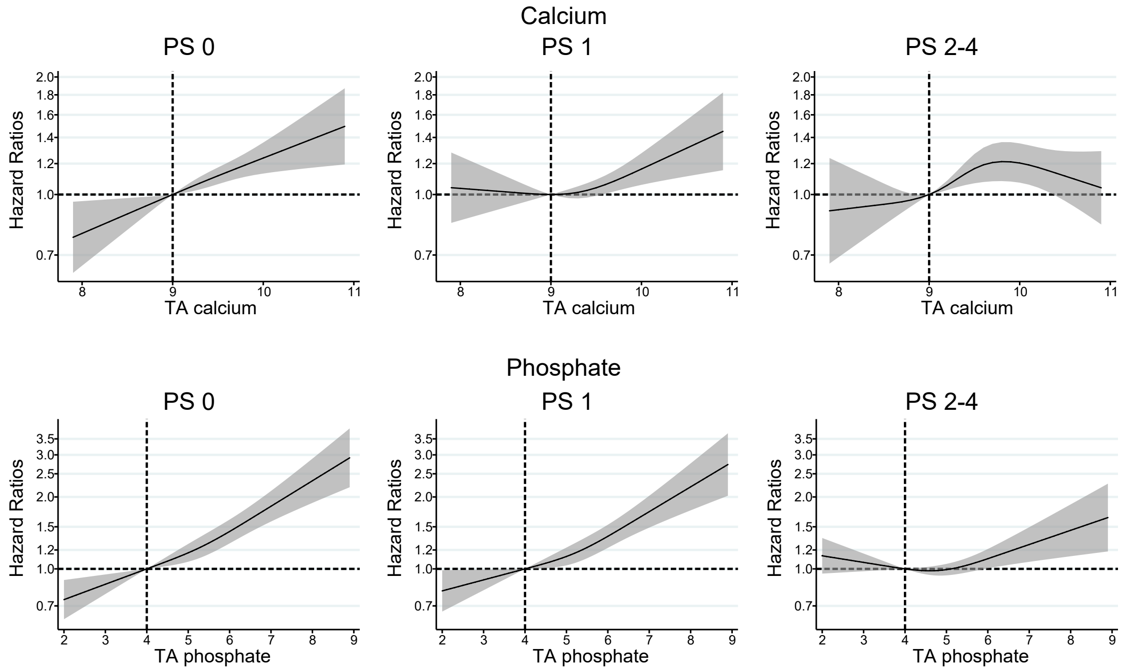

Figure S6. Relationship of time-averaged (TA) serum calcium or phosphate levels with mortality due to heart failure in patients stratified by performance status (PS).

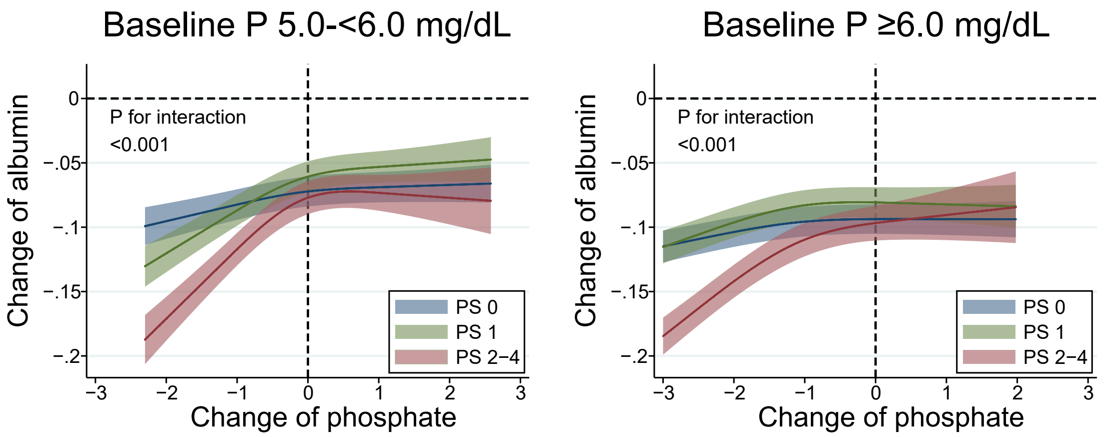

Figure S7. Relationship between changes in serum phosphate levels and those in serum albumin levels in patients stratified by performance status (PS).

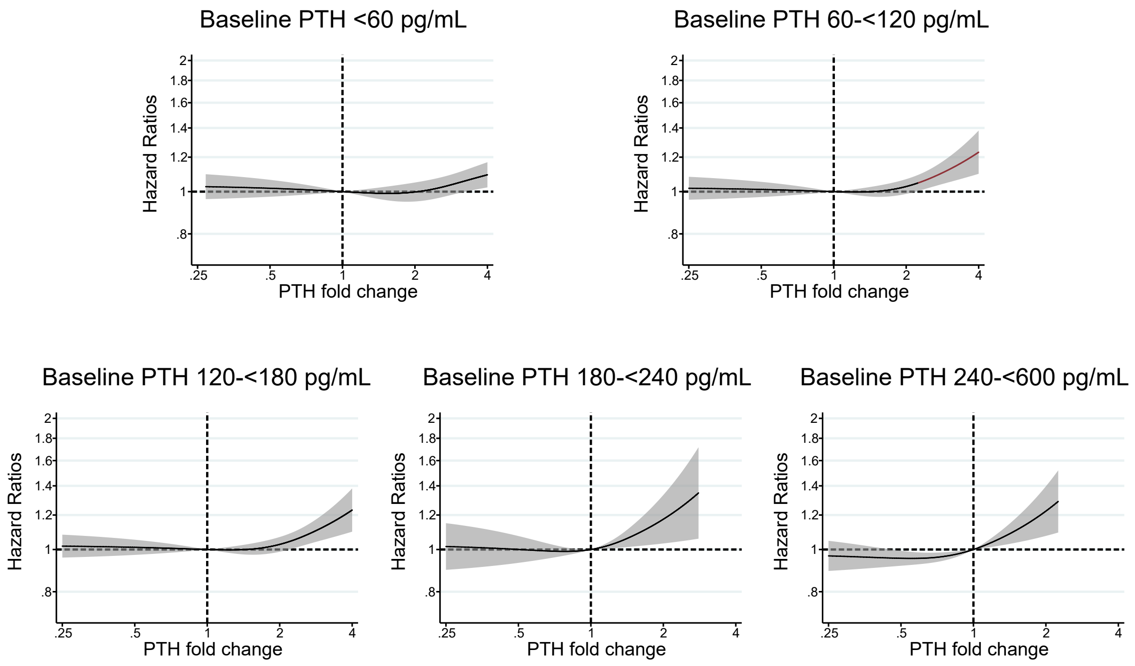

Figure S8. Relationship between percent changes in serum intact parathyroid hormone (PTH) levels from baseline values and all-cause mortality.
